# What do we really know about micronutrient supplementation and risk for upper respiratory infection?

**DOI:** 10.1136/bmjgh-2020-004896

**Published:** 2021-01-19

**Authors:** Sydne Newberry

**Affiliations:** Southern California Evidence-based Practice Center, The RAND Corporation, Santa Monica, California, USA

**Keywords:** health education and promotion

Dietary nutrition researchers have been trying to understand how nutrition and nutrients influence immune function for a long time—over 60 years, judging by the dates of some of the earliest published research papers on nutrition and immune function.

It should come as no surprise then that this interest has reached a fever pitch as governments, healthcare systems and especially public health systems worldwide face what is likely the biggest health challenge in most of our lifetimes and probably over a century—preventing, treating and averting long-term consequences of SARS-CoV-2 (COVID-19) infection. Within a month or two of acknowledgements that COVID-19 had reached pandemic proportions, it seems, the mainstream media was already reporting conflicting untested evidence about the possibility that vitamin D or C supplements might play a role in preventing or mitigating infection, and some reputable clinicians were asserting that even in the absence of evidence, perhaps it would be prudent just to take some extra vitamin D.^1^

It is in this context that Abioye and colleagues^2^ decided to systematically review the literature on the role of dietary vitamin and mineral supplements in preventing or shortening the course of upper respiratory infections (URIs). The bottom line is that much of the research, which dates back decades, was poorly designed and reported, and the evidence for benefits is weak, confirming the assertions of several other relatively recent reviews on nutrients and immune function.^3 4^ Nevertheless, the evidence, as weak as it is, could easily encourage false hopes, potentially improving nothing but dietary supplement manufacturers’ own bottom line.

It is with the hope of averting a media frenzy and the inevitable rush to the supplement aisle or website that I write this editorial. But more importantly—I hope—perhaps the review and this editorial will help design better studies and avoid past mistakes in trying to assess the role of micronutrient status and dietary and supplemental micronutrients in the risk for, natural course of, and treatment and long-term outcomes of COVID-19. In that spirit, I call out some of the major flaws of this research, using the population, interventions, comparators, outcomes, timing, setting, and study design framework of study characteristics.

***Populations.*** The studies included in the review by Abioye *et al* enrolled populations that we know from other research have a wide range of baseline health and micronutrient status and usual intakes and, in the case of vitamin D, sun exposure,^5^ but few of the studies actually reported or accounted for baseline status in analysing the results of supplementation. As the authors noted, trials in low-income and middle-income countries were more likely to observe beneficial effects of vitamin D supplements on risk for developing URIs. Types of URIs also varied between viral and bacterial. Although not certain, this difference could potentially have contributed to heterogeneity within and among studies.

***Interventions.*** Immune function is affected by the total diet, especially by intake of protein and calories. In low-income and middle-income countries, the likelihood of macronutrient inadequacy among those with inadequate micronutrient status cannot be dismissed. This is one of the challenges of identifying the role of individual micronutrient status in human immune function, and it could help explain the findings of Abioye *et al* that micronutrient supplements have at best borderline significant impacts on the risk for URIs. The studies on multivitamin combinations were too small in number and heterogeneous in intervention design to combine meaningfully.

Few if any of the studies Abioye *et al* identified assessed achieved nutrient status following supplementation, despite adequate biomarkers of status, especially for vitamin D. However, another major concern when pooling studies of vitamin D supplementation is that the methods used to assess vitamin D have changed over time, such that older and newer studies really should not be pooled. The Vitamin D Standardization Program has published a manual that enables comparison of study findings across time and assay methods.^6^

Another concern with the studies is the wide range of dosing across the studies included in the review, from physiological to clearly pharmacological doses. Part of the concern involves verifying the actual nutrient contents of the supplements. However, a bigger concern is that dietary supplements delivered at supraphysiological doses can have effects that are totally unrelated to the physiological roles of the nutrients.

***Comparators.*** Few of the original studies included in the review described the process used to randomise participants to intervention and control groups, how these allocations were concealed, and what processes if any were used to prevent contamination and to ensure control groups were not taking personal supplements.

***Outcomes.*** Many of the included studies relied on self-reported symptoms of onset and end of symptoms, although some did conduct clinical verification (which the review did not further describe). In addition, some studies used active challenge to induce infection, whereas others relied on passive exposure: these studies should not have been pooled. Of greater concern, none of the studies apparently assessed safety. Dietary supplements, especially those that deliver supraphysiological doses, can be associated with a spectrum of adverse events, some serious, due to the micronutrient itself or to toxic contaminants. even when administered for relatively short periods of time. This brings up the next issue: time courses of treatment and follow-up.

***Time courses for follow-up.*** The time courses of treatment and follow-up varied widely across studies. Since baseline and follow-up nutrient status were seldom reported, who is to say what an appropriate duration would be for treatment and follow-up?

***Setting.*** The differences in study setting, as discussed previously, would be of particular concern, partly due to differences in the incidence of micronutrient deficiencies and in the case of vitamin D due to differences in sun exposure.

***Study design.*** The Abioye *et al* review included only randomised control trials (RCTs), excluding observational studies. Presumably, RCTs distribute potential individual confounding factors randomly across intervention groups. However, because the studies were generally low quality--with few to no studies assessing baseline vitamin or mineral status, none controlling for age or comorbidities, geographic areas and doses ranging widely, and some studies actively challenging participants and others waiting for passive infection--pooling such heterogeneous studies is not likely to provide terribly useful answers, and although this may be a minor concern, few studies confirmed the actual pathogen responsible for infection.

In conclusion, based on the quality of the included studies, the inconsistency and imprecision of findings across studies, and the small effect sizes, the strength of the evidence supporting any beneficial effects of individual or combination micronutrient supplements is low at best, although the review did not attempt to assess the strength of evidence for any of the micronutrients. Given this likely low strength of evidence, the concern today should be focused on ensuring that food insecure populations, especially those at higher risk for viral infections because of age and/or comorbidities, be guaranteed access to nutritionally adequate, food-based diets to prevent or mitigate the risk for COVID-19 or any infectious condition.
